# The Italian telephone-based Verbal Fluency Battery (t-VFB): standardization and preliminary clinical usability evidence

**DOI:** 10.3389/fpsyg.2022.963164

**Published:** 2022-08-03

**Authors:** Edoardo Nicolò Aiello, Alice Naomi Preti, Veronica Pucci, Lorenzo Diana, Alessia Corvaglia, Chiara Barattieri di San Pietro, Teresa Difonzo, Stefano Zago, Ildebrando Appollonio, Sara Mondini, Nadia Bolognini

**Affiliations:** ^1^Ph.D. Program in Neuroscience, School of Medicine and Surgery, University of Milano-Bicocca, Monza, Italy; ^2^Fondazione IRCCS Ca’ Granda Ospedale Maggiore Policlinico, University of Milan, Milan, Italy; ^3^Department of Philosophy, Sociology, Pedagogy and Applied Psychology, University of Padova, Padua, Italy; ^4^Human Inspired Technology Research Centre, University of Padova, Padua, Italy; ^5^Neuropsychological Laboratory, IRCCS Istituto Auxologico Italiano, Milan, Italy; ^6^Department of Biomedical and Clinical Sciences Luigi Sacco, University of Milan, Milan, Italy; ^7^Department of Clinical and Experimental Sciences, University of Brescia, Brescia, Italy; ^8^Department of Neurosciences, Biomedicine and Movement Sciences, University of Verona, Verona, Italy; ^9^Neurology Section, School of Medicine and Surgery, University of Milano-Bicocca, Monza, Italy; ^10^Department of Psychology, University of Milano-Bicocca, Milan, Italy

**Keywords:** verbal fluency, tele-neuropsychology, executive functioning, language, telephone-based

## Abstract

**Background:**

This study aimed at standardizing and providing preliminary evidence on the clinical usability of the Italian telephone-based Verbal Fluency Battery (t-VFB), which includes phonemic (t-PVF), semantic (t-SVF) and alternate (t-AVF) verbal fluency tasks.

**Methods:**

Three-hundred and thirty-five Italian healthy participants (HPs; 140 males; age *range* = 18–96 years; education *range* = 4–23 years) and 27 individuals with neurodegenerative or cerebrovascular diseases were administered the t-VFB. Switch number and cluster size were computed *via* latent semantic analyses. HPs underwent the telephone-based Mental State Examination (MMSE) and Backward Digit Span (BDS). Construct validity, factorial structure, internal consistency, test-retest and inter-rater reliability and equivalence with the in-person Verbal Fluency tasks were assessed. Norms were derived *via* Equivalent Scores. Diagnostic accuracy against clinical populations was assessed.

**Results:**

The majority of t-VFB scores correlated among each other and with the BDS, but not with the MMSE. Switch number correlated with t-PVF, t-SVF, t-AVF scores, whilst cluster size with the t-SVF and t-AVF scores only. The t-VFB was underpinned by a mono-component structure and was internally consistent (Cronbach’s α = 0.91). Test-retest (ICC = 0.69–0.95) and inter-rater reliability (ICC = 0.98–1) were optimal. Each t-VFB test was statistically equivalent to its in-person version (equivalence bounds yielding a *p* < 0.05). Education predicted all t-VFB scores, whereas age t-SVF and t-AVF scores and sex only some t-SVF scores. Diagnostic accuracy against clinical samples was optimal (AUC = 0.81–0.86).

**Discussion:**

The t-VFB is a valid, reliable and normed telephone-based assessment tool for language and executive functioning, equivalent to the in-person version; results show promising evidence of its diagnostic accuracy in neurological populations.

## Introduction

Verbal fluency tasks are renowned measures of both language (i.e., lexical retrieval) and executive functioning (i.e., executive control over selective attention, inhibition, set-shifting and self-monitoring). These tests usually require to produce, within a given time (usually of 60 s), as many words as possible starting with a given letter (phonemic verbal fluency, PFV) or belonging to a given category (semantic verbal fluency, SVF) (Whiteside et al., 2016; Aita et al., 2018). There is also an alternate verbal fluency test (AVF), which requires to generate words by continuously alternating between phonemically and semantically cued ones, thus assessing set-shifting abilities (Costa et al., 2014).

As not requiring either visual or physical supports, verbal fluency tasks have been widely adopted within telephone-based cognitive assessment (Rapp et al., 2012; Bunker et al., 2017; Marceaux et al., 2019), both as screening measures and within comprehensive neuropsychological batteries (Carlew et al., 2020).

Telephone-based cognitive assessment is relevant to clinical (e.g., for not-viable/-preferable access to clinics due to logistical, geographical, economical or health safety reasons; Christodoulou et al., 2016; Caze et al., 2020; De Cola et al., 2020; Soldati et al., 2021) and experimental telemedicine (e.g., population-based/epidemiological studies, decentralized clinical trials and prevention campaigns; Crooks et al., 2005; Yaari et al., 2006; Herr and Ankri, 2013). However, function-specific telephone-based tests often do not meet rigorous statistical-methodological standards, especially within the Italian context (Zanin et al., 2022). Moreover, so far, standardized telephone-based cognitive assessment tools in Italy are limited to screening tests for the assessment of global cognitive status (Aiello et al., 2022a,d,e).

Telephone-based adaptations of existing tests require *ad hoc* norms, psychometrics and diagnostics even if their administration and scoring are comparable to their in-person versions, since unknown sources of systematic error variance may affect scores due to the different administration setting (Parlar et al., 2020; Fox-Fuller et al., 2021; Postal et al., 2021; Zanin et al., 2022). More specifically, threats to the equivalence between in-person and telephone-based measures have been identified in a lack of control over the testing environment (e.g., due to sources of distractions or facilitations), technical issues (e.g., telephone line instability) and interpersonal aspects (e.g., the absence of a face-to-face interaction), all these factors being leading confounders especially in examinees with sensory-motor, cognitive or behavioral disorders (Martin-Khan et al., 2010; Binng et al., 2020; Booth et al., 2021; Hunter et al., 2021). Moreover, the lack of *ad hoc* standardizations has been highlighted also by clinicians as a barrier to remote cognitive assessment procedures (Rochette et al., 2021).

Such issues likewise apply to telephone-based verbal fluency tasks, which have been recognized as not completely comparable to in-person ones (Hunter et al., 2021). Nevertheless, no *ad hoc* standardizations of telephone-based verbal fluency tasks have been provided so far.

Given the above premises, the present study aimed at standardizing, for the Italian population, a telephone-based Verbal Fluency Battery (t-VFB) comprising PVF, SVF, and AVF tasks, as well as at preliminarily assessing its clinical usability in individuals with cerebrovascular or neurodegenerative diseases.

## Materials and methods

### Participants

Three-hundred and thirty-five Italian healthy participants (HPs) from different regions of Italy represented the normative sample. HPs were recruited through both Authors’ personal acquaintances and advertising at the University of Milano-Bicocca and University of Padova. HPs had no history of (1) neurological/psychiatric disorders, (2) active psychotropic medications, (3) uncompensated/severe general-medical conditions and (4) uncorrected hearing deficits.

Twenty-seven outpatients with neurological diseases were consecutively recruited at two neuropsychology services in Northern Italy. A clinical neurological diagnosis formulated by a neurologist constituted the inclusion criteria for clinical groups. Diagnoses were posed according to current diagnostic criteria and based on neurological, neuroradiological and neuropsychological examinations. Exclusion criteria for patients were: (1) severe medical-general conditions in the acute phase; (2) severe behavioral impairment that would have undermined compliance; (3) uncorrected hearing deficits. Ten participants had ischemic/hemorrhagic stroke causing unilateral, cortical or subcortical hemispheric lesion (5 with right-sided hemispheric lesion, and 5 with left-sided lesion). Six individuals presented with hypokinetic, extra-pyramidal disorders: 3 had Parkinson’s disease (Postuma et al., 2015), whereas 3 had atypical parkinsonisms (Levin et al., 2016). Six individuals had small vessel disease (Shi and Wardlaw, 2016). Four had a mixed, atrophic-vascular dementia (Zekry et al., 2002). Diagnoses of stroke, small vessel disease and mixed dementia were supported by computerized tomography or magnetic resonance imaging, whereas those of extra-pyramidal disorders by single-photon emission computerized tomography of the dopamine transporter. All patients underwent, for clinical purposes, a neuropsychological battery comprising measures of overall cognitive efficiency (Mini-Mental State Examination (Measso et al., 1993), attention (digit cancellation test, Spinnler and Tognoni, 1987; Trail-Making Test, Siciliano et al., 2019), executive functions (Frontal Assessment Battery Appollonio et al., 2005; Raven Colored Progressive Matrices Basso et al., 1987; phonemic and alternate verbal fluency Costa et al., 2014; Stroop test Caffarra et al., 2002), language (Token Test, Spinnler and Tognoni, 1987; Boston Naming Test, Goodglass et al., 1983; semantic verbal fluency, Costa et al., 2014), verbal and visuo-spatial memory (Babcock test, Novelli et al., 1986; forward/backward digit and Corsi span, Monaco et al., 2013) and visuo-spatial/constructional abilities (Clock Drawing Test, Caffarra et al., 2011; design copy, Spinnler and Tognoni, 1987). Additionally, stroke patients with a left-sided hemispheric lesion underwent a comprehensive language (Esame Neuropsicologico per l’Afasia; Capasso and Miceli, 2001) and limb apraxia assessment (ideomotor praxis, De Renzi et al., 1980; ideative praxis, De Renzi and Lucchelli, 1988), whereas in those with a right-sided damage extra-personal and personal hemispatial neglect was assessed (Bells test, Vallar et al., 1994; letter cancellation test, Vallar et al., 1994; Apple Cancellation Test, Mancuso et al., 2015; line bisection test, Nichelli et al., 1989; Fluff test, Cocchini et al., 2001; Comb and Razor test, Zoccolotti et al., 1992).

Medical history for all participants was collected through a semi-structured interview.

The study received ethical approval by the Committees of the University of Milano-Bicocca, Milano (ID: RM-2021-382, 19/02/2021), the University of Padova, Padua (ID: 4107, 19/02/2021) and IRCCS Istituto Auxologico Italiano, Milano (ID: 25C122, 18/05/2021). Informed consent was acquired from every participant. Data collection for HPs started in March 2021 and ended in January 2022, whereas that for clinical populations started in July 2021 and ended in January 2022.

### Materials

The t-VFB comprises the telephone-based versions of 3 verbal fluency subtests: the t-PVF, t-SVF, and t-AVF subtests standardized by Costa et al.’s (2014), which were administered over the telephone. The t-PVF requires to generate as many words as possible beginning with letters “F,” “A,” and “S,” within a 60”-timespan each. The t-SVF require to generate semantic category exemplars: “Color,” “Animal,” and “Fruit” (60 s for each category). The t-AVF requires to continuously alternate letter-cued words with category-cued words as follows: “A/Color,” “F/Animal,” “S/Fruit”; again, each trial lasted 60 s. Following the administration procedure for the in-person version of Costa et al.’s (2014) battery, the three verbal fluency subtests were administered consecutively in the following order: t-PVF, t-SVF, and t-AVF; according to the original work, PVF and SVF tasks must precede the AVF subtest in order to calculate the Composite Shifting Index (CSI), i.e., a measure of the cost of shifting from phonemic/semantic (single-cued) to alternating (double-cued) fluency. As in the original work by Costa et al. (2014), participants were instructed not to produce proper nouns, place names, numbers or inflected words with the same suffix. For each trial, the number of words generated in 60 s was recorded. Performance score in each subtest is computed as the sum of the number of words generated in all trials belonging to that specific subtest. A t-CSI was also computed as follows: t-AVF/[(t-PVF + t-SVF)/2]. The t-CSI thus addresses the words generated in all three subtests, and it reflects the shifting cost for passing from the single fluency subtests to the alternate one.

The protocol of the t-VFB is reported in Supplementary Material 1. The t-VFB protocol was conceptualized and approved without reservation by an Author board comprising a neurologist (IA), six researchers with expertise in neuropsychology and psychometrics (SZ, SM, NB, ENA, VP, and LD).

To assess convergent validity, HPs were remotely administered the Italian telephone-based Mini-Mental State Examination (Itel-MMSE) (Metitieri et al., 2001; Aiello et al., 2022b) and a telephone version of the backward digit span (BDS) task (Monaco et al., 2013). The BDS encompasses both the longer sequence recalled, measuring working memory capacity (BDS-WM), and the total number of recalled sequences (BDS-T), measuring sustained attention (Pasotti et al., 2022).

### Procedures

Before telephone-based testing, all participants underwent a detailed sound-check to ensure a good quality of the call, as well as and a brief training focused on those actions required for the executions of telephone-based tasks (Supplementary Material 2). For each participant, a caregiver/cohabitee was required to preliminarily ensure that no distractions or facilitations were present within the administration setting. The same person was asked to confirm the correctness of the address information provided by the participant during the spatial orientation task of the Itel-MMSE.

To test between-modality equivalence, a subgroup of 47 HPs were also administered in-person verbal fluency tasks either before (*N* = 23) or after (*N* = 24) a 48-h distance from telephonic testing, to rule out possible carry-over effects.

Eighteen HPs were re-tested with the t-VFB after 14 days from the baseline for test-retest reliability, whereas 27 protocols were simultaneously scored by two independent raters for inter-rater reliability.

All the above sub-samples for validity/reliability analyses were randomly selected from the whole normative sample.

Data were collected by either licensed or trainee psychologists who first underwent a thorough training.

### Statistical analyses

SPSS 27 (IBM Corp., 2020), R 4.1.0^1^ and jamovi 1.6.23^2^ were adopted to analyze data.

Convergent validity was explored through either Pearson’s or Spearman correlations for normally and non-normally distributed data, respectively (skewness and kurtosis values < |1| and | 3|, respectively; Kim, 2013).

Intra-class correlations were adopted to examine test-retest and inter-rater reliability. Internal consistency and factorial structure were tested *via* Cronbach’s α and a Principal Component Analysis (PCA), respectively.

Verbal fluency performance relies on both language (i.e., lexical-semantic abilities) and executive functioning (Whiteside et al., 2016; Aita et al., 2018). According to Troyer et al. (1997), a valid measure of lexical-semantic integrity derived from verbal fluency is the number of circumscribable clusters of semantically-related words (cluster size), while a measure of executive functioning is the frequency of transitions between these clusters (number of switches). Accordingly, to provide further construct validity evidence, the number of switches and cluster size were computed relying on measures of semantic relatedness derived *via* a Latent Semantic Analysis, which is a natural language processing statistical model (Landauer and Dumais, 1997).

A two one-sided test (TOST) procedure for dependent samples (Lakens, 2017) was adopted for testing the equivalence between telephone-based and in-person verbal fluency tasks. The TOST procedure regards a between-mean effect size as equivalent to 0 if it falls within the upper and lower equivalence bounds and distances from them at *p* < 0.05.

Norms were derived through the Equivalent Score (ES) approach (Capitani and Laiacona, 2017; Aiello and Depaoli, 2022). Accordingly, clinical judgments were allowed by identifying outer and inner tolerance limits (oTL; iTL), as well as ES thresholds, on stepwise regression-adjusted scores. The ES scales allows to draw clinical judgments as follows: ES = 0 (adjusted scores ≤ oTL) → impaired; ES = 1→ borderline; ES = 2→ “low-end” normal; ES = 3/4→ normal.

Diagnostic accuracy of the t-VFB was tested *via* receiver-operating characteristics analyses by addressing scores of the whole clinical group against the whole normative sample.

### Power analyses

The minimum sample size for reliability and validity analyses in HPs were set at *N*≈20 and *N*≈80, respectively, pursuantly to Hobart et al.’s (2012) recommendations on psychometric measurement in neurology.

According to qualitative guidelines delivered by Kyriazos (2018), a sample of 100 subjects was deemed as sufficient for running the PCA.

Sample size estimation for the TOST procedure, as run through the R package *TOSTER*^3^ (Lakens, 2017), yielded a minimum of *N* = 44 pairs with a 90% power, α = 0.05 and upper and lower equivalence bounds of -0.5 and 0.5, respectively.

For norm derivation, an estimated *N* = 287 was deemed sufficient to detect a small-to-medium effect size (*f*^2^ = 0.05) with a 90% power and a type-I error rate of 5% within a multiple regression model (*df*_numerator_ = 3) through the R package *pwr*^4^.

The minimum sample sizes for the normative and clinical groups for diagnostic accuracy analyses were set at *N* = 19 and *N* = 190, respectively, in accordance with Obuchowski’s (2005) procedures for a single-test ROC analysis and *via easyROC*^5^, by addressing a case-control allocation ratio of 10, AUC = 0.7, 1-β = 0.9 and α = 0.05.

## Results

Table 1 shows the stratification of the normative sample, and Table 2 summarizes HPs’ demographic data and test scores.

**TABLE 1:** Sample stratification for age, education, and sex.

M/F	Age						
Education	30≤	31–45	46–60	61–70	71–80	≥81	Total
5≤	0/0	0/0	0/0	1/3	1/2	6	3/11
6–8	5/0	2/2	8/13	4/2	4/3	2/3	25/23
9–12	1/1	0/1	2/11	4/6	2/2	1/1	10/22
13–16	34/28	8/11	20/39	4/13	1/3	0/1	67/95
≥17	12/19	4/5	9/11	7/7	2/0	1/2	35/44
Total	52/48	14/19	39/74	20/31	10/10	5/13	140/195

**TABLE 2:** Demographic and cognitive data of the normative sample.

***N***		335
**Age (years)**		48.42 ± 18.84 (18–96)
**Sex (M/F)**		140/195
**Education (years)**		13.42 ± 3.78 (4–23)
*N* for Italian regions	North Italy	(81.7%)
	Center Italy	(4.7%)
	South Italy	(13.6%)
*N* for occupation	Predominantly manual	75 (22.4%)
	Manual/clerical	78 (23.3%)
	Predominantly clerical	163 (48.7%)
Itel-MMSE (*N* = 252)		21.58 ± 0.82 (17–22)
BDS-WM (*N* = 266)		4.78 ± 1.22 (0–6)
BDS-T (*N* = 266)		4.97 ± 1.98 (0–8)
t-VFB (*N* = 335)	t-PVF-A	13.1 ± 4.83 (2–28)
	t-PVF-F	14.98 ± 4.9 (3–30)
	t-PVF-S	14.52 ± 5.08 (2–34)
	t-PVF (total)	42.6 ± 13.48 (13–85)
	t-SVF-Colors	15.74 ± 3.99 (2–34)
	t-SVF-Animals	22.9 ± 6.94 (0–43)
	t-SVF-Fruits	16.88 ± 4.49 (0–31)
	t-SVF (total)	55.52 ± 13.12 (3–84)
	t-AVF-A/Colors	13.62 ± 5.1 (2–26)
	t-AVF-F/Animals	14.13 ± 5.12 (0–28)
	t-AVF-S/Fruits	14.39 ± 4.93 (0–28)
	t-AVF (total)	42.11 ± 13.66 (4–74)
	t-CSI	0.86 ± 0.21 (0.1–2.22)

N, number of participants; M, male; F, female; Itel-MMSE, Italian telephone-based Mini-Mental State Examination; BDS-WM, backward digit span working memory; BDS-t, backward digit span total; t-VFB, telephone-based Verbal Fluency Battery; t-PVF, telephone-based phonemic verbal fluency; t-SVF, telephone-based semantic verbal fluency; t-AVF, telephone-based alternate verbal fluency; t-CSI, telephone-based Cognitive Shifting Index.

Acceptability rate was 100%.

Convergence against the other telephone-based measures (Itel-MMSE and BDS) is reported in Supplementary Table 1. At α_adjusted_ = 0.001, the vast majority of t-VFB measures were associated with BDS-T and BDS-WM scores; by contrast, the Itel-MMSE was only associated with the t-AVF and t-CSI, but not to t-PVF and t-SVF scores.

When putting into relation all t-VFB measures among each other, significant, moderate-to-high correlations were detected [0.33 ≤ *r*_*s*_(335) ≤ 0.92; all *p*s < 0.001] among t-PVF, t-SVF, and t-AVF, whereas, as to the t-CSI, significant associations where only detected with t-AVF scores [*r*_*s*_(335) ≥ 0.54; all *p*s < 0.001]. At α_adjusted_ = 0.006, the number of switches was strongly related with total t-PVF, t-SVF and t-AVF scores [0.71 ≤ *r*_*s*_(138) ≤ 0.74; all *p*s < 0.001], whereas cluster size was related to the t-SVF and t-AVF [both *r*_*s*_(138) = -0.32, *p* < 0.001], but not to t-PVF [*r*_*s*_(138) = -0.16, *p* = 0.067]. The t-CSI was not associated with either the number switches or cluster size (*p* ≥ 0.009).

The PCA revealed a mono-component structure underpinning t-PVF, t-SVF, and t-AVF scores (59.7% of variance explained; loading *range* = 0.65–0.83), here named “linguistically mediated executive functioning.” Internal consistency for such scores was excellent (Cronbach’s α = 0.91).

Inter-rater reliability was excellent for all t-VFB total scores: t-PVF: ICC = 1; t-SVF: ICC = 0.98; t-AVF: ICC = 0.98. Test-retest reliability was optimal for the t-PVF (ICC = 0.95) and t-AVF (ICC = 0.89), and good for the t-SVF (ICC = 0.69).

The TOST procedure revealed across-modality equivalence for all verbal fluency total scores: PVF: telephone-based: 42.6 ± 11.2 vs. in-person 41.7 ± 11, *t*(46) = 0.63, *p* = 0.533, upper and lower equivalence bounds both yielding a *p* ≤ 0.01; SVF: telephone-based: 55.9 ± 11.6 vs. in-person 53.7 ± 11.7, *t*(46) = -1.45, *p* = 0.154, upper and lower equivalence bounds both yielding a *p* < 0.05; AVF: telephone-based: 43.5 ± 11.1 vs. in-person 43.5 ± 12, *t*(46) = -0.01, *p* = 0.988, upper and lower equivalence bounds both yielding a *p* ≤ 0.001; CSI: telephone-based:0.89 ± 0.17 vs. in-person 0.91 ± 0.2, *t*(46) = 0.68, *p* = 0.501, upper and lower equivalence bounds both yielding a *p* ≤ 0.01.

Education predicted all t-VFB measures (*p*s ≤ 0.029), age the t-SVF, t-AVF and t-CSI (*p* ≤ 0.001), whereas sex predicted only t-SVF-colors (*p* = 0.006) and t-SVF-fruits scores (*p* < 0.001), with females performing better than males. Adjustment equations, TLS and ES thresholds are reported in Table 3; adjustment grids are reported in Supplementary Tables 2–4, whereas an automated adjusted sheet in Supplementary Material 3.

**TABLE 3:** Adjustment equations and Equivalent Scores of the telephone-based Verbal Fluency Battery (t-VFB).

**Measure**	**Adjustment equations**						
t-PVF-F	AS = RS-0.581857*(edu-13.41791)						
t-PVF-A	AS = RS-0.546721*(edu-13.41791)						
t-PVF-S	AS = RS-3.459879*(sqrt(edu)-3.621862)						
t-PVF (Total)	AS = RS-11.19203*(sqrt(edu)-3.621862)						
t-SVF-Colors	AS = RS + 0.000008*((age^3)-165449.256716)-4.337454*(log10(edu)-1.106749) + 0.549597 if M; -0.549597 if F						
t-SVF-Animals	AS = RS + 0.000008*((age^3)-165449.256716)-14.77632*(log10(edu)-1.106749)						
t-SVF-Fruits	AS = RS + 0.000007*((age^3)-165449.256716)-2.030476*(sqrt(edu)-3.621862) + 1.191156 if M; -1.191156 if F						
t-SVF (Total)	AS = RS + 0.000023*((age^3)-165449.256716)-26.77201*(log10(edu)-1.106749)						
t-AVF-A/Colors	AS = RS + 0.000765*((age^2)-2698.337313)-12.67585*(log10(edu)-1.106749)						
t-AVF-F/Animals	AS = RS + 0.000731*((age^2)-2698.337313)-14.25557*(log10(edu)-1.106749)						
t-AVF-S/Fruits	AS = RS + 0.00001*((age^3)-165449.256716)-10.65985*(log10(edu)-1.106749)						
t-AVF (Total)	AS = RS + 0.002323*((age^2)-2698.337313)-37.46202*(log10(edu)-1.106749)						
t-CSI	AS = RS + 0.000029*((age^2)-2698.337313) + 0.818169*((1/edu)-0.083184)						

	**Equivalent scores**						
	**oTL**	**iTL**	**0**	**1**	**2**	**3**	**4**

t-PVF-F	6.24	8.92	≤6.24	6.25–10.15	10.16–12.33	12.34–14.83	≥14.84
t-PVF-A	5.23	7.04	≤5.23	5.24–7.78	7.79–10.49	10.50–13.32	≥13.33
t-PVF-S	5.69	8.06	≤5.69	5.70–9.06	9.07–11.75	11.76–14.55	≥14.56
t-PVF (Total)	21.18	26.05	≤21.18	21.19–28.18	28.19–35.05	35.06–42.77	≥42.78
t-SVF-Colors	9.80	10.91	≤9.80	9.81–11.62	11.63–13.81	13.82–15.51	≥15.52
t-SVF-Animals	11.25	14.39	≤11.25	11.26–15.40	15.41–18.81	18.82–22.69	≥22.70
t-SVF-Fruits	9.36	11.08	≤9.36	9.37–11.90	11.91–14.07	14.08–16.81	≥16.82
t-SVF (Total)	35.09	39.23	≤35.09	35.10–41.26	41.27–47.84	47.85–56.33	≥56.34
t-AVF-A/Colors	5.46	7	≤5.46	5.47–8.01	8.02–11.11	11.12–13.92	≥13.93
t-AVF-F/Animals	5.75	7.93	≤5.75	5.76–8.98	8.99–11.49	11.50–14.29	≥14.30
t-AVF-S/Fruits	6.47	8.37	≤6.47	6.48–9.43	9.44–11.83	11.84–14.37	≥14.38
t-AVF (Total)	19.60	25.91	≤19.60	19.61–29.27	29.28–35.66	35.67–42.36	≥42.37
t-CSI	0.48	0.60	≤0.48	0.49–0.66	0.67–0.76	0.77–0.86	≥0.87

PVF, Phonemic Verbal Fluency; SVF, Semantic Verbal Fluency; AVF, Alternate Verbal Fluency; CSI, Composite Shifting Index; AS, Adjusted Score; RS, Raw Score; edu, years of education; age, years of age; sqrt, square root; iTL, inner Tolerance Limit; oTL, outer Tolerance Limit. Adjustment grids and a sheet for the automated computation of ASs are provided in Supplementary Material.

Table 4 reports demographic data and test scores of individuals with neurological diseases. When comparing the whole clinical group against the normative sample, each t-VFB task yielded excellent accuracy: t-PVF: AUC = 0.81, *SE* = 0.05, CI 95% [0.71, 0.91]; t-SVF: AUC = 0.81, *SE* = 0.04, CI 95% [0.73, 0.9]; t-AVF: AUC = 0.86, *SE* = 0.03, CI 95% [0.8, 0.93].

**TABLE 4:** Patients’ demographic, clinical, and cognitive data.

	Total	EPD	SVD	Stroke (5 left-sided, 5 right-sided lesion)	MD
*N*	27	6	6	10	5
Sex (M/F)	13/14	4/2	2/4	5/5	2/3
Age (years)	71.74 ± 12.38 (38–91)	76 ± 5.06 (70–82)	81.33 ± 7.97 (68–91)	61.7 ± 12.91 (38–78)	75.2 ± 8.79 (60–82)
Education (years)	13.67 ± 4.57 (5–18)	14 ± 5.18 (5–18)	14.5 ± 3.94 (8–18)	13.9 ± 4.51 (8–18)	11.8 ± 5.63 (5–18)
MMSE (*N* = 25)	26.4 ± 3.11 (20–30)	29 ± 1.27 (27–30)	23.33 ± 1.75 (21–26)	27.5 ± 2.51 (23–30)	25.2 ± 3.56 (20–30)
t-PVF (*N* = 21)	27.33 ± 11.92 (11–59)	–	30.33 ± 13.28 (18–50)	28.2 ± 12.03 (17–59)	22 ± 10.65 (11–39)
t-SVF (*N* = 14)	40.21 ± 11 (17–56)	–	–	43.2 ± 6.88 (33–54)	32.75 ± 1668 (17–56)
t-AVF (*N* = 23)	21.48 ± 12.03 (6-44)	30.67 ± 11.36 (18-44)	16.33 ± 7.53 (10-26)	20.4 ± 12.47 (6–36)	–
t-CSI (*N* = 10)	–	–	–	0.55 ± 0.3 (0.2–1.09)	–

N, number of participants; M, male; F, female; EPD, extra-pyramidal disorder; SVD, small vessel disease; MD, mixed dementia; MMSE, Mini-Mental State Examination; t-PVF, telephone-based phonemic verbal fluency; t-SVF, telephone-based semantic verbal fluency; t-AVF, telephone-based alternate verbal fluency; t-CSI, telephone-based Cognitive Shifting Index.

## Discussion

The present study provides Italian practitioners and researchers with a standardized, telephone-based set of verbal fluency tests (t-VFB), along with clinical usability evidence in participants affected with neurological conditions of different etiologies. As providing modality-specific norms, psychometrics and diagnostics of verbal fluency tasks to be administered over the telephone, this work is unprecedented both within the Italian and international literature, enriching the range of telephone-based cognitive tests available in Italy (Aiello et al., 2022a,d,c) by meeting statistical-methodological requirements suggested for telephone-based tools-related standardizations (Zanin et al., 2022). In this last respect, it is remarkable that the present telephone-based version of verbal fluency tests showed to be statistically equivalent to the in-person versions by Costa et al. (2014), this supporting, within longitudinal assessments, a flexible use of the t-VFB in combination with the in-person version normed by Costa et al. (2014). However, this finding should not lead users to address, for the t-VFB, the same norms, psychometrics and diagnostics of in-person verbal fluency tests. Indeed, in order to avoid distortion in test scores, such information needs to be derived through *ad hoc* standardization studies specific to the telephonic modality, consistently to international guidelines on remote cognitive testing (Postal et al., 2021; Zanin et al., 2022). Users of the t-VFB could nonetheless safely compare, within longitudinal assessments, the ESs yielded from its administration to those yielded by the administration of Costa et al.’s (2014) battery, since the 5-points ES scale allows to compare score at different tests (Capitani and Laiacona, 2017; Aiello and Depaoli, 2022).

As coming with norms separately for each task and subtask, the t-VFB represents a flexible tool as to its administration. Moreover, the t-VFB presents with a solid factorial structure, optimal construct validity, internal consistency, test-retest, and inter-rater reliability. This work also provides promising, albeit preliminary, diagnostic accuracy evidence of the t-VFB in individuals with cerebrovascular and neurodegenerative diseases. The construct validity of the t-VFB is also supported by its association with renowned, fine-grained measures of both language- and executive-related verbal fluency underpinnings, i.e., the cluster size and the number of switches, respectively (Troyer et al., 1997). Indeed, all t-VFB measures were related to the number of switches, i.e., the executive component common to all verbal fluency tasks (Whiteside et al., 2016), whereas only the t-SVF and t-AVF, which rely more on language functions as compared to PVF, correlated with cluster size, that is a proper language measure reflecting the integrity of the lexical-semantic component (Shao et al., 2014; Aita et al., 2018).

Data on demographic effects are overall in line with those reported by Costa et al. (2014): t-SVF, t-AVF, and t-CSI scores were predicted by both age and education, whereas the t-PVF only by the latter. However, in Costa et al. (2014), sex predicted all PVF, SVF, and AVF scores, whilst, within the present work, it was only predictive of certain t-SVF subtest scores. This discrepancy is surprising, considering that the samples of both studies had a similar number of females and males, i.e., 56% of females in Costa et al. (2014), whereas 58% in the present study. Sex differences in healthy individuals on verbal fluency have been previously reported, although current evidence is still unclear and varied from report to report (Heister, 1982; Gauthier et al., 2009). Nevertheless, previous findings appear to sufficiently converge as to a slight female advantage at least for PVF (Capitani et al., 1998).

As to the limitations, it should be noted that the present findings about the clinical usability of the t-VFB refer to a small sample of participants with neurological diseases of different etiology. Future studies should extend and verify the present results in larger and homogeneous clinical cohorts featured by executive and/or language disorders that could influence verbal fluency performance (Suárez-González et al., 2021). Within such clinical usability studies, post-test probabilities (positive and negative predictive values and likelihood ratios), responsiveness and reliable change need also to be assessed (Aiello et al., 2022c). Moreover, equivalence of the t-VFB with its in-person version needs to be documented also in neurological populations.

In conclusions, the t-VFB shows to be a valid, reliable, diagnostically accurate and normed telephone-based cognitive tool for the assessment of linguistically mediated executive functions. The t-VFB is equivalent to in-person verbal fluency tasks and shows promising evidence of clinical usability in individuals with cerebrovascular or neurodegenerative diseases.

## Data availability statement

The datasets presented in this study can be found in online repositories. The names of the repository/repositories and accession number(s) can be found below: https://osf.io/75nsd/.

## Ethics statement

The studies involving human participants were reviewed and approved by the Ethics Committee of the University of Milano-Bicocca, Milano (ID: RM-2021-382, 19/02/2021), the University of Padova, Padua (ID: 4107, 19/02/2021) and IRCCS Istituto Auxologico Italiano, Milano (ID: 25C122, 18/05/2021). The patients/participants provided their written informed consent to participate in this study.

## Author contributions

EA and NB: conceptualization. EA, AP, VP, LD, AC, TD, SZ, IA, SM, and NB: data curation. EA, AP, VP, LD, and CB: formal analysis. NB: funding acquisition. EA, VP, LD, and AC: Investigation. EA and CB: Methodology. SZ, IA, SM, and NB: re-sources. TD, SZ, IA, and SM: supervision. EA, AP, VP, LD, CB, and NB: writing–original draft. EA, AP, VP, LD, AC, CB, TD, SZ, IA, SM, and NB: writing–review and editing. All authors have read and agreed to the published version of the manuscript.

## Footnotes

1
https://cran.r-project.org/

2
https://www.jamovi.org/

3
https://cran.r-project.org/web/packages/TOSTER/TOSTER.pdf

4
https://cran.r-project.org/web/packages/pwr/pwr.pdf

5
http://www.biosoft.hacettepe.edu.tr/easyROC/
