# Supplementary material for: The Italian telephone-based Verbal Fluency Battery (t-VFB): standardization and preliminary clinical usability evidence
Source: Front Psychol. 2022 Aug 3;13:963164. doi: 10.3389/fpsyg.2022.963164 (PMC9384842; doi:10.3389/fpsyg.2022.963164)
Supplement: Supplementary file 2 [file Data_Sheet_2.docx]

**Telephone-based Verbal Fluecy Battery (t-VFB) (versione**

[Tratto da: Costa *et al.*, 2014]

| **Fonemiche:** “In un minuto di tempo, dovrà dirmi tutte le parole che le vengono in mente che iniziano con una data lettera dell’alfabeto che le dirò. Non potrà dirmi nomi propri di persona o di luoghi, e numeri.” ***Note:*** *no parole con lo stesso prefisso e declinate (ad es., “andare, andavo, andato” = 1 pt.; “automobile, autostrada”= 2 pt.).* | | “F”:  [__] | |
| --- | --- | --- | --- |
|  |  | “A”:  [__] | |
|  |  | “S”:  [__] | |
| **Semantiche:** “In un minuto di tempo, dovrà dirmi tutte le parole che le vengono in mente che appartengono ad una data categoria che le dirò. Non potrà dirmi nomi propri di persona o di luoghi e numeri.” ***Note:*** *no diminutivi.*  **Tot.: [____]** | | “Colori”:  [__] | |
|  |  | “Animali”:  [__] | |
|  |  | “Frutti”:  [__] | |
| **Alternate:** “In un minuto di tempo, dovrà alternare, quante più volte, una parola che inizia con una determinata lettera dell’alfabeto, ed una che appartiene ad una data categoria. Ad esempio: se le dico “P” e “verdure”, lei dovrà dirmi: “ponte”-“carota”; “parrucchiere”-“insalata”, e così via. Non potrà dirmi nomi propri di persona o di luoghi e numeri.” ***Note:*** *no parole declinate/coniugate*.  **Attribuzione del punteggio**: 2 pt. per ogni alternanza corretta; 0 pt. per un’alternanza incompleta (non è possibile assegnare 1 pt.)  **Tot.: [____]** | | “A”-“Colori”:  [__] | |
|  |  | “F”-“Animali”:  [__] | |
|  |  | “S”-“Frutti”:  [__] | |
| **Composite Shifting Index:** Alternate/[(Fonemiche+Semantiche)/2]=__/[(__+__/2)]=__ | | | |
|  | **PG** | **PC** | **PE** |
| **t-PVF-F** |  |  |  |
| **t-PVF-A** |  |  |  |
| **t-PVF-S** |  |  |  |
| **t-PVF** |  |  |  |
| **t-SVF-Colors** |  |  |  |
| **t-SVF-Animals** |  |  |  |
| **t-SVF-Fruits** |  |  |  |
| **t-SVF** |  |  |  |
| **t-AVF-A/Colors** |  |  |  |
| **t-AVF-F/Animals** |  |  |  |
| **t-AVF-S/Fruits** |  |  |  |
| **t-AVF** |  |  |  |
| **t-CSI** |  |  |  |

**Telephone-based Verbal Fluecy Battery (t-VFB; translated, English version)**

[From: Costa *et al.*, 2014]

| **Phonemic:** “You have one minute to name as many words as possible beginning with a given letter. Proper names (persons and places) and numbers are not allowed.” ***Notes:*** *derived words with the same prefix are not allowed (e.g., “go, goes, gone” = 1 pt.; “neurologist, neurodegenerative”= 2 pts.).* | | “F”:  [__] | |
| --- | --- | --- | --- |
|  |  | “A”:  [__] | |
|  |  | “S”:  [__] | |
| **Semantic:** “You have one minute to name as many words as possible belonging to a given category. Proper names (persons and places) and numbers are not allowed”*.* ***Note:*** *diminutives are not allowed.*  **Tot.: [____]** | | “Colors”:  [__] | |
|  |  | “Animals”:  [__] | |
|  |  | “Fruits”:  [__] | |
| **Alternate:** “You have one minute to alternate, as many times as possible, a word beginning with a given letter and a word belonging to a given category. For example: if I say “P” and “Vegetables”, you should say: “Page-Carrot”; “Pain-Beetroot” and so on. Proper names (persons and places) and numbers are not allowed. ***Note:*** *derived words are not allowed..*  .  **Scoring**: 2 pts. for each pair of correctly alternated words; 0 pts. for each incomplete pair (1 pt. cannot be assigned).  **Tot.: [____]** | | “A”-“Colors”:  [__] | |
|  |  | “F”-“Animals”:  [__] | |
|  |  | “S”-“Fruits”:  [__] | |
| **Composite Shifting Index:** Alternate/[(Phonemic+Semantic)/2]=__/[(__+__/2)]=__ | | | |
|  | **RS** | **CS** | **ES** |
| **t-PVF-F** |  |  |  |
| **t-PVF-A** |  |  |  |
| **t-PVF-S** |  |  |  |
| **t-PVF** |  |  |  |
| **t-SVF-Colors** |  |  |  |
| **t-SVF-Animals** |  |  |  |
| **t-SVF-Fruits** |  |  |  |
| **t-SVF** |  |  |  |
| **t-AVF-A/Colors** |  |  |  |
| **t-AVF-F/Animals** |  |  |  |
| **t-AVF-S/Fruits** |  |  |  |
| **t-AVF** |  |  |  |
| **t-CSI** |  |  |  |
