# Supplementary material for: The Italian telephone-based Verbal Fluency Battery (t-VFB): standardization and preliminary clinical usability evidence
Source: Front Psychol. 2022 Aug 3;13:963164. doi: 10.3389/fpsyg.2022.963164 (PMC9384842; doi:10.3389/fpsyg.2022.963164)
Supplement: Supplementary file 3 [file Data_Sheet_3.docx]

*Setting* sperimentale e *Sound-check* (versione Italiana)

[Brandt *et al.*, 1988; Christodoulou *et al.*, 2016]

**Informazioni generali**

Presenza di una persona terza: sì□ no□

*Setting* scevro da distrazioni: sì□ no□

*Setting* scevro da fonti di suggerimento: sì□ no□

**Informazioni del partecipante da richiedere alla persona terza:**

nome: _______ cognome: _______

*Dati relativi al luogo in cui il partecipante si trova durante la sessione di* baseline*:*

via/piazza: ___________ numero civico: ____ città: ___________ provincia: ___________ C.A.P.: ___________ regione: ___________ stato: ___________

**Informazioni sul dispositivo**

Telefono: cellulare □ telefono di casa □ altro (specificare): ___________

Tastiera: *smartphone* (*touch-screen*) □ telefono con tasti rialzati □ tastierino □ visualizzabile/visualizzato □

□ altro (specificare): ___________

Vivavoce: attivato □ non attivabile/non attivato □

*Fornire indicazioni adeguate per l’attivazione del vivavoce e la visualizzazione del tastierino (qualora necessarie).*

***Sound-check***

a. “Per garantire la buona riuscita dell’intervista, ci piacerebbe verificare la qualità del suono della telefonata. Dica il più velocemente possibile “undici-dodici-tredici-quattordici-quindici”. Ha notato eco, ritardo od altre distorsioni nel suono?”

*a.1. Completare i campi sottostanti in base a quanto risposto dal partecipante (0=nessun problema rilevato; 3=eco+ritardo+altre difficoltà).*

eco □ ritardo □ altre difficoltà (indicare quali):_________________________________________

Punteggio: ____/3

*a.2. Completare i campi sottostanti in base alla percezione dell’intervistatore (0=nessun problema rilevato; 3=eco+ritardo+altre difficoltà).*

eco □ ritardo □ altre difficoltà (indicare quali):_________________________________________

Punteggio: ____/3

b. “Dica “verde”. Batta un colpo sul tavolo. Ha notato eco, ritardo od altre distorsioni nel suono?”

*b.1. Completare i campi sottostanti in base a quanto risposto dal partecipante(0=nessun problema rilevato; 3=eco+ritardo+altre difficoltà).*

eco □ ritardo □ altre difficoltà (indicare quali):_________________________________________

Punteggio: ____/3

*b.2. Completare i campi sottostanti in base alla percezione dell’intervistatore (0=nessun problema rilevato; 3=eco+ritardo+altre difficoltà).*

eco □ ritardo □ altre difficoltà (indicare quali):_________________________________________

Punteggio: ____/3

c. “Adesso batterò tre volte sul tavolo. Ha sentito bene i colpi? Ha notato eco, ritardo od altre distorsioni nel suono?”

*c.1. Completare i campi sottostanti in base a quanto risposto dal partecipante(0=nessun problema rilevato; 3=eco+ritardo+altre difficoltà).*

eco □ ritardo □ altre difficoltà (indicare quali):_________________________________________

Punteggio: ____/3

*c.2. Completare i campi sottostanti in base alla percezione dell’intervistatore (0=nessun problema rilevato; 3=eco+ritardo+altre difficoltà).*

eco □ ritardo □ altre difficoltà (indicare quali):_________________________________________

Punteggio: ____/3

d. “Adesso le dirò delle coppie di parole; mi dica se sono uguali o diverse: «tasso-tasso», «frana-frana», «vera-vera», «palla-palla», «sfoglia-foglia», «pollo-bollo», «fino-vino», «sonno-sonno», passo-pasto», «messo-nesso».

Punteggio: >2 errori=2; 2 errori=1; ≤1 errore=0: ___

Punteggio totale al *sound-check* (a.1.+a.2.+b.1.+b.2.+c.1.+c.2.+ d): ____/20

Experimental Setting and Sound-check (translated, English version)

[Brandt *et al.*, 1988; Christodoulou *et al.*, 2016]

**General Information**

Presence of a third person: yes□ no□

Setting free from sources of distractions: yes□ no□

Setting free from sources of suggestions yes□ no□

**Participant’s data to be obtained from the third person:**

First name: _______ Surname: _______

*Participant’s location during the baseline session:*

Street: ___________ House number: ____ City: ___________ Province: ___________ Postal Code: ___________ Region: ___________ Country: ___________

**Information about the device:**

Phone: mobile □ landline phone □ other (specify): ___________

Keypad: smartphone (touch-screen) □ landline phone keypad □ mobile phone keypad □ can be visualized by activating the speakerphone □ other (specify): ___________

Speakerphone: active □ not active/can’t be activated □

*If necessary, provide adequate instructions for activating the speakerphone and displaying the keypad*

**Sound-check**

a. “To ensure the success of the interview, we have to check the sound quality of this call. Say "undici-dodici-tredici-quattordici-quindici" as quickly as possible. Have you noticed any echo, delay or other distortions in the sound?”

*a.1. Fill out the fields below according to the participant’s answer (0 = no problem detected; 3 = echo + delay + other difficulties).*

echo □ delay □ other difficulties (specify):_____________________________________

Score: ____/3

*a.2. Fill out the fields below according to the examiner’s perception (0 = no problem detected; 3 = echo + delay + other difficulties).*

echo □ delay □ other difficulties (specify):_________________________________________

Score: ____/3

b. “Say “verde”. Tap once on the table. Have you noticed any echo, delay or other distortions in the sound?”

*b.1. Fill out the fields below according to the participant’s answer (0 = no problem detected; 3 = echo + delay + other difficulties).*

echo □ delay □ other difficulties (specify):_____________________________________

Score: ____/3

*b.2. Fill out the fields below according to the examiner’s perception (0 = no problem detected; 3 = echo + delay + other difficulties)*

echo □ delay □ other difficulties (specify):_____________________________________

Score: ____/3

c. “I am going to tap three times on the table. Have you heard the taps? Have you noticed any echo, delay or other distortions in the sound?”

*c.1. Fill out the fields below according to the participant’s answer (0 = no problem detected; 3 = echo + delay + other difficulties).*

echo □ delay □ other difficulties (specify):_____________________________________

Score: ____/3

*c.2. Fill out the fields below according to the examiner’s perception (0 = no problem detected; 3 = echo + delay + other difficulties).*

echo □ delay □ other difficulties (specify):_________________________________________

Score: ____/3

d. “I am going to tell you some word pairs; please, tell me if they are the same or different: «tasso-tasso», «frana-frana», «vera-vera», «palla-palla», «sfoglia-foglia», «pollo-bollo», «fino-vino», «sonno-sonno», passo-pasto», «messo-nesso».

Score: >2 errors=2; 2 errors=1; ≤1 error=0: ___

Total sound-check score (a.1.+a.2.+b.1.+b.2.+c.1.+c.2.+ d): ____/
