# Supplementary material for: The Italian telephone-based Verbal Fluency Battery (t-VFB): standardization and preliminary clinical usability evidence
Source: Front Psychol. 2022 Aug 3;13:963164. doi: 10.3389/fpsyg.2022.963164 (PMC9384842; doi:10.3389/fpsyg.2022.963164)
Supplement: Supplementary file 4 [file Table_1.docx]

**Supplementary Table 1.** Spearman’s correlation coefficients between t-VFB and construct validity measures.

|  | Itel-MMSE | BDS-T | BDS-WM |
| --- | --- | --- | --- |
| t-PVF-F | *r_s_*(252)=.15* | *r_s_*(266)=.29** | *r_s_*(266)=.29** |
| t-PVF-A | *r_s_*(252)=.09**†** | *r_s_*(266)=.25** | *r_s_*(266)=.25** |
| t-PVF-S | *r_s_*(252)=.08**†** | *r_s_*(266)=.29** | *r_s_*(266)=.29** |
| t-PVF | *r_s_*(252)=.11**†** | *r_s_*(266)=.3** | *r_s_*(266)=.3** |
| t-SVF-Colors | *r_s_*(252)=.11**†** | *r_s_*(266)=.26** | *r_s_*(266)=.25** |
| t-SVF-Animals | *r_s_*(252)=.16* | *r_s_*(266)=.22** | *r_s_*(266)=.24** |
| t-SVF-Fruits | *r_s_*(252)=.17* | *r_s_*(266)=.21** | *r_s_*(266)=.19* |
| t-SVF | *r_s_*(252)=.17* | *r_s_*(266)=.26** | *r_s_*(266)=.26** |
| t-AVF-A/Colors | *r_s_*(252)=.23** | *r_s_*(266)=.33** | *r_s_*(266)=.33** |
| t-AVF-F/Animals | *r_s_*(252)=.21** | *r_s_*(266)=.32** | *r_s_*(266)=.33** |
| t-AVF-S/Fruits | *r_s_*(252)=.26** | *r_s_*(266)=.31** | *r_s_*(266)=.31** |
| t-AVF | *r_s_*(252)=.26** | *r_s_*(266)=.35** | *r_s_*(266)=.35** |
| t-CSI | *r_s_*(252)=.21** | *r_s_*(266)=.19* | *r_s_*(266)=.2** |
| Notes. †non-significant; *significant at α=.05; **significant at α_adjusted_=.00. t-PVF=telephone-based phonemic verbal fluency; t-SVF=telephone-based semantic verbal fluency; t-AVF=telephone-based alternate verbal fluency; t-CSI=telephone-based cognitive shifting index; -t=total; Itel-MMSE=Italian telephone-based of the Mini-Mental State Examination; BDS-WM= backward digit span working memory; BDS-t=backward digit span total. | | | |
