# Supplementary material for: The Italian telephone-based Verbal Fluency Battery (t-VFB): standardization and preliminary clinical usability evidence
Source: Front Psychol. 2022 Aug 3;13:963164. doi: 10.3389/fpsyg.2022.963164 (PMC9384842; doi:10.3389/fpsyg.2022.963164)
Supplement: Supplementary file 5 [file Table_2.docx]

**Supplementary Table 2.** Adjustment grids for the telephone-based phonemic verbal fluency (t-PVF)

|  | **Education** | | | | | |
| --- | --- | --- | --- | --- | --- | --- |
|  | **5** | **8** | **11** | **13** | **16** | **18** |
|  | **t-PVF-F** | | | | | |
|  | 4.90 | 3.15 | 1.41 | 0.24 | -1.50 | -2.67 |
|  | **t-PVF-A** | | | | | |
|  | 4.60 | 2.96 | 1.32 | 0.23 | -1.41 | -2.51 |
|  | **t-PVF-S** | | | | | |
|  | 4.79 | 2.75 | 1.06 | 0.06 | -1.31 | -2.15 |
|  | **t-PVF (total)** | | | | | |
|  | 15.51 | 8.88 | 3.42 | 0.18 | -4.23 | -6.95 |

**Notes.** Adjustment factors have been extracted from the adjustment equations (see the body of the article) and do not always reflect empirical co-occurrences.
