# Supplementary material for: The Italian telephone-based Verbal Fluency Battery (t-VFB): standardization and preliminary clinical usability evidence
Source: Front Psychol. 2022 Aug 3;13:963164. doi: 10.3389/fpsyg.2022.963164 (PMC9384842; doi:10.3389/fpsyg.2022.963164)
Supplement: Supplementary file 6 [file Table_3.docx]

**Supplementary Table 3.** Adjustment grids for the telephone-based semantic verbal fluency (t-VFB).

|  | **Education** | | | | | |
| --- | --- | --- | --- | --- | --- | --- |
|  | **5** | **8** | **11** | **13** | **16** | **18** |
| **Age** | **t-SVF-Colors (Females)** | | | | | |
| **35** | 0.24 | -0.65 | -1.25 | -1.56 | -1.95 | -2.17 |
| **40** | 0.41 | -0.48 | -1.08 | -1.39 | -1.78 | -2.01 |
| **45** | 0.62 | -0.26 | -0.86 | -1.18 | -1.57 | -1.79 |
| **50** | 0.90 | 0.01 | -0.59 | -0.90 | -1.30 | -1.52 |
| **55** | 1.23 | 0.34 | -0.26 | -0.57 | -0.96 | -1.19 |
| **60** | 1.62 | 0.74 | 0.14 | -0.18 | -0.57 | -0.79 |
| **65** | 2.09 | 1.21 | 0.61 | 0.29 | -0.10 | -0.32 |
| **70** | 2.64 | 1.75 | 1.15 | 0.84 | 0.45 | 0.23 |
| **75** | 3.27 | 2.39 | 1.79 | 1.47 | 1.08 | 0.86 |
| **80** | 3.99 | 3.11 | 2.51 | 2.19 | 1.80 | 1.58 |
| **85** | 4.81 | 3.92 | 3.32 | 3.01 | 2.62 | 2.40 |
| **90** | 5.73 | 4.84 | 4.24 | 3.93 | 3.54 | 3.31 |
|  | **t-SVF-Colors (Males)** | | | | | |
| **35** | 1.34 | 0.45 | -0.15 | -0.46 | -0.85 | -1.08 |
| **40** | 1.51 | 0.62 | 0.02 | -0.29 | -0.68 | -0.91 |
| **45** | 1.72 | 0.84 | 0.24 | -0.08 | -0.47 | -0.69 |
| **50** | 1.99 | 1.11 | 0.51 | 0.19 | -0.20 | -0.42 |
| **55** | 2.33 | 1.44 | 0.84 | 0.53 | 0.13 | -0.09 |
| **60** | 2.72 | 1.84 | 1.24 | 0.92 | 0.53 | 0.31 |
| **65** | 3.19 | 2.31 | 1.71 | 1.39 | 1.00 | 0.78 |
| **70** | 3.74 | 2.85 | 2.25 | 1.94 | 1.55 | 1.33 |
| **75** | 4.37 | 3.48 | 2.88 | 2.57 | 2.18 | 1.96 |
| **80** | 5.09 | 4.21 | 3.61 | 3.29 | 2.90 | 2.68 |
| **85** | 5.91 | 5.02 | 4.42 | 4.11 | 3.72 | 3.49 |
| **90** | 6.83 | 5.94 | 5.34 | 5.03 | 4.64 | 4.41 |
|  | **t-SVF-Animals** | | | | | |
| **35** | 5.04 | 2.03 | -0.01 | -1.09 | -2.42 | -3.18 |
| **40** | 5.21 | 2.20 | 0.15 | -0.92 | -2.25 | -3.01 |
| **45** | 5.43 | 2.41 | 0.37 | -0.70 | -2.03 | -2.79 |
| **50** | 5.70 | 2.69 | 0.64 | -0.43 | -1.76 | -2.52 |
| **55** | 6.03 | 3.02 | 0.97 | -0.10 | -1.43 | -2.19 |
| **60** | 6.43 | 3.41 | 1.37 | 0.30 | -1.03 | -1.79 |
| **65** | 6.90 | 3.88 | 1.84 | 0.77 | -0.57 | -1.32 |
| **70** | 7.45 | 4.43 | 2.39 | 1.31 | -0.02 | -0.77 |
| **75** | 8.08 | 5.06 | 3.02 | 1.95 | 0.61 | -0.14 |
| **80** | 8.80 | 5.78 | 3.74 | 2.67 | 1.33 | 0.58 |
| **85** | 9.61 | 6.60 | 4.56 | 3.48 | 2.15 | 1.39 |
| **90** | 10.53 | 7.52 | 5.47 | 4.40 | 3.07 | 2.31 |
|  | **t-SVF-Fruits (Females)** | | | | | |
| **35** | 0.76 | -0.44 | -1.43 | -2.02 | -2.82 | -3.31 |
| **40** | 0.91 | -0.29 | -1.28 | -1.87 | -2.67 | -3.16 |
| **45** | 1.10 | -0.10 | -1.09 | -1.68 | -2.48 | -2.97 |
| **50** | 1.34 | 0.14 | -0.85 | -1.44 | -2.24 | -2.73 |
| **55** | 1.63 | 0.43 | -0.56 | -1.15 | -1.95 | -2.45 |
| **60** | 1.98 | 0.77 | -0.22 | -0.80 | -1.61 | -2.10 |
| **65** | 2.39 | 1.18 | 0.19 | -0.39 | -1.19 | -1.69 |
| **70** | 2.87 | 1.66 | 0.67 | 0.08 | -0.72 | -1.21 |
| **75** | 3.42 | 2.21 | 1.22 | 0.64 | -0.16 | -0.66 |
| **80** | 4.05 | 2.85 | 1.85 | 1.27 | 0.47 | -0.03 |
| **85** | 4.76 | 3.56 | 2.57 | 1.98 | 1.18 | 0.69 |
| **90** | 5.57 | 4.36 | 3.37 | 2.79 | 1.99 | 1.49 |
|  | **t-SVF -Fruits (Males)** | | | | | |
| **35** | 3.15 | 1.94 | 0.95 | 0.37 | -0.43 | -0.93 |
| **40** | 3.29 | 2.09 | 1.10 | 0.51 | -0.29 | -0.78 |
| **45** | 3.48 | 2.28 | 1.29 | 0.70 | -0.10 | -0.59 |
| **50** | 3.72 | 2.52 | 1.53 | 0.94 | 0.14 | -0.35 |
| **55** | 4.01 | 2.81 | 1.82 | 1.23 | 0.43 | -0.06 |
| **60** | 4.36 | 3.16 | 2.16 | 1.58 | 0.78 | 0.28 |
| **65** | 4.77 | 3.57 | 2.58 | 1.99 | 1.19 | 0.69 |
| **70** | 5.25 | 4.05 | 3.05 | 2.47 | 1.67 | 1.17 |
| **75** | 5.80 | 4.60 | 3.61 | 3.02 | 2.22 | 1.73 |
| **80** | 6.43 | 5.23 | 4.24 | 3.65 | 2.85 | 2.36 |
| **85** | 7.15 | 5.94 | 4.95 | 4.37 | 3.56 | 3.07 |
| **90** | 7.95 | 6.75 | 5.76 | 5.17 | 4.37 | 3.88 |
|  | **t-SVF (Total)** | | | | | |
| **35** | 8.10 | 2.63 | -1.07 | -3.01 | -5.43 | -6.80 |
| **40** | 8.58 | 3.12 | -0.58 | -2.53 | -4.94 | -6.31 |
| **45** | 9.21 | 3.74 | 0.04 | -1.90 | -4.32 | -5.69 |
| **50** | 9.99 | 4.52 | 0.82 | -1.12 | -3.54 | -4.91 |
| **55** | 10.94 | 5.47 | 1.77 | -0.17 | -2.59 | -3.95 |
| **60** | 12.08 | 6.62 | 2.91 | 0.97 | -1.44 | -2.81 |
| **65** | 13.43 | 7.96 | 4.26 | 2.32 | -0.10 | -1.47 |
| **70** | 15.00 | 9.54 | 5.83 | 3.89 | 1.48 | 0.11 |
| **75** | 16.81 | 11.35 | 7.65 | 5.71 | 3.29 | 1.92 |
| **80** | 18.89 | 13.42 | 9.72 | 7.78 | 5.36 | 3.99 |
| **85** | 21.24 | 15.77 | 12.07 | 10.13 | 7.71 | 6.34 |
| **90** | 23.88 | 18.41 | 14.71 | 12.77 | 10.35 | 8.99 |

**Notes.** Adjustment factors have been extracted from the adjustment equations (see the body of the article) and do not always reflect empirical co-occurrences.
