# Supplementary material for: The Italian telephone-based Verbal Fluency Battery (t-VFB): standardization and preliminary clinical usability evidence
Source: Front Psychol. 2022 Aug 3;13:963164. doi: 10.3389/fpsyg.2022.963164 (PMC9384842; doi:10.3389/fpsyg.2022.963164)
Supplement: Supplementary file 7 [file Table_4.docx]

**Supplementary Table 4.** Adjustment grids for Alternate Verbal Fluency (AVF) and the Composite Shifting Index (CSI)

|  | **Education** | | | | | |
| --- | --- | --- | --- | --- | --- | --- |
|  | **5** | **8** | **11** | **13** | **16** | **18** |
| **Age** | **t-AVF-A/Colors** | | | | | |
| **35** | 4.04 | 1.45 | -0.30 | -1.22 | -2.36 | -3.01 |
| **40** | 4.33 | 1.74 | -0.01 | -0.93 | -2.07 | -2.72 |
| **45** | 4.65 | 2.07 | 0.31 | -0.61 | -1.75 | -2.40 |
| **50** | 5.02 | 2.43 | 0.68 | -0.24 | -1.39 | -2.03 |
| **55** | 5.42 | 2.83 | 1.08 | 0.16 | -0.98 | -1.63 |
| **60** | 5.86 | 3.27 | 1.52 | 0.60 | -0.54 | -1.19 |
| **65** | 6.34 | 3.75 | 2.00 | 1.08 | -0.07 | -0.71 |
| **70** | 6.85 | 4.27 | 2.51 | 1.59 | 0.45 | -0.20 |
| **75** | 7.41 | 4.82 | 3.07 | 2.15 | 1.00 | 0.36 |
| **80** | 8.00 | 5.41 | 3.66 | 2.74 | 1.60 | 0.95 |
| **85** | 8.63 | 6.04 | 4.29 | 3.37 | 2.23 | 1.58 |
| **90** | 9.30 | 6.71 | 4.96 | 4.04 | 2.90 | 2.25 |
|  | **t-AVF-F/Animals** | | | | | |
| **35** | 4.74 | 1.83 | -0.15 | -1.18 | -2.47 | -3.19 |
| **40** | 5.01 | 2.10 | 0.13 | -0.91 | -2.19 | -2.92 |
| **45** | 5.32 | 2.41 | 0.44 | -0.59 | -1.88 | -2.61 |
| **50** | 5.67 | 2.76 | 0.79 | -0.25 | -1.53 | -2.26 |
| **55** | 6.05 | 3.14 | 1.17 | 0.14 | -1.15 | -1.88 |
| **60** | 6.47 | 3.56 | 1.59 | 0.56 | -0.73 | -1.46 |
| **65** | 6.93 | 4.02 | 2.05 | 1.01 | -0.27 | -1.00 |
| **70** | 7.42 | 4.51 | 2.54 | 1.51 | 0.22 | -0.51 |
| **75** | 7.95 | 5.04 | 3.07 | 2.04 | 0.75 | 0.02 |
| **80** | 8.52 | 5.61 | 3.64 | 2.60 | 1.32 | 0.59 |
| **85** | 9.12 | 6.21 | 4.24 | 3.21 | 1.92 | 1.19 |
| **90** | 9.76 | 6.85 | 4.88 | 3.85 | 2.56 | 1.83 |
|  | **t-AVF-S/Fruits** | | | | | |
| **35** | 3.12 | 0.95 | -0.53 | -1.30 | -2.26 | -2.81 |
| **40** | 3.33 | 1.16 | -0.32 | -1.09 | -2.05 | -2.60 |
| **45** | 3.60 | 1.43 | -0.05 | -0.82 | -1.78 | -2.33 |
| **50** | 3.94 | 1.77 | 0.29 | -0.48 | -1.44 | -1.99 |
| **55** | 4.36 | 2.18 | 0.71 | -0.07 | -1.03 | -1.57 |
| **60** | 4.85 | 2.68 | 1.20 | 0.43 | -0.53 | -1.08 |
| **65** | 5.44 | 3.26 | 1.79 | 1.02 | 0.05 | -0.49 |
| **70** | 6.12 | 3.95 | 2.47 | 1.70 | 0.74 | 0.19 |
| **75** | 6.91 | 4.74 | 3.26 | 2.49 | 1.53 | 0.98 |
| **80** | 7.81 | 5.64 | 4.16 | 3.39 | 2.43 | 1.88 |
| **85** | 8.83 | 6.66 | 5.18 | 4.41 | 3.45 | 2.90 |
| **90** | 9.98 | 7.81 | 6.33 | 5.56 | 4.60 | 4.05 |
|  | **t-AVF (Total)** | | | | | |
| **35** | 11.85 | 4.21 | -0.97 | -3.69 | -7.07 | -8.99 |
| **40** | 12.72 | 5.08 | -0.10 | -2.82 | -6.20 | -8.12 |
| **45** | 13.71 | 6.07 | 0.88 | -1.83 | -5.21 | -7.13 |
| **50** | 14.82 | 7.17 | 1.99 | -0.73 | -4.11 | -6.02 |
| **55** | 16.04 | 8.39 | 3.21 | 0.49 | -2.89 | -4.81 |
| **60** | 17.37 | 9.72 | 4.54 | 1.83 | -1.55 | -3.47 |
| **65** | 18.82 | 11.18 | 5.99 | 3.28 | -0.10 | -2.02 |
| **70** | 20.39 | 12.74 | 7.56 | 4.84 | 1.47 | -0.45 |
| **75** | 22.07 | 14.43 | 9.25 | 6.53 | 3.15 | 1.23 |
| **80** | 23.88 | 16.23 | 11.05 | 8.33 | 4.95 | 3.03 |
| **85** | 25.79 | 18.14 | 12.96 | 10.25 | 6.87 | 4.95 |
| **90** | 27.82 | 20.18 | 15.00 | 12.28 | 8.90 | 6.98 |
|  | **t-CSI** | | | | | |
| **35** | 0.05 | -0.01 | -0.04 | -0.05 | -0.06 | -0.07 |
| **40** | 0.06 | 0.00 | -0.03 | -0.04 | -0.05 | -0.05 |
| **45** | 0.08 | 0.01 | -0.01 | -0.02 | -0.04 | -0.04 |
| **50** | 0.09 | 0.03 | 0.00 | -0.01 | -0.02 | -0.03 |
| **55** | 0.11 | 0.04 | 0.02 | 0.00 | -0.01 | -0.01 |
| **60** | 0.12 | 0.06 | 0.03 | 0.02 | 0.01 | 0.00 |
| **65** | 0.14 | 0.08 | 0.05 | 0.04 | 0.03 | 0.02 |
| **70** | 0.16 | 0.10 | 0.07 | 0.06 | 0.05 | 0.04 |
| **75** | 0.18 | 0.12 | 0.09 | 0.08 | 0.07 | 0.06 |
| **80** | 0.20 | 0.14 | 0.11 | 0.10 | 0.09 | 0.08 |
| **85** | 0.23 | 0.17 | 0.14 | 0.13 | 0.11 | 0.11 |
| **90** | 0.25 | 0.19 | 0.16 | 0.15 | 0.14 | 0.13 |

**Notes.** Adjustment factors have been extracted from the adjustment equations (see the body of the article) and do not always reflect empirical co-occurrences.
